# Mueller matrix imaging for collagen scoring in mice model of pregnancy

**DOI:** 10.1038/s41598-021-95020-8

**Published:** 2021-08-02

**Authors:** Hee Ryung Lee, Ilyas Saytashev, Vinh Nguyen Du Le, Mala Mahendroo, Jessica Ramella-Roman, Tatiana Novikova

**Affiliations:** 1 LPICM, CNRS, Ecole polytechnique, IP Paris, 91128 Palaiseau, France; 2 Department of Biomedical Engineering, College of Engineering and Computing, Florida International University, 10555 West Flagler Street, Miami, FL 33174 USA; 3 Department of Obstetrics and Gynecology, University of Texas Southwestern Medical Center, Dallas, Texas 75390 USA; 4 Department of Ophthalmology, Herbert Wertheim College of Medicine, Florida International University, 11200 SW 8th Street, Miami, FL 33199 USA

**Keywords:** Biophotonics, Imaging and sensing, Polarization microscopy

## Abstract

Preterm birth risk is associated with early softening of the uterine cervix in pregnancy due to the accelerated remodeling of collagen extracellular matrix. Studies of mice model of pregnancy were performed with an imaging Mueller polarimeter at different time points of pregnancy to find polarimetric parameters for collagen scoring. Mueller matrix images of the unstained sections of mice uterine cervices were taken at day 6 and day 18 of 19-days gestation period and at different spatial locations through the cervices. The logarithmic decomposition of the recorded Mueller matrices mapped the depolarization, linear retardance, and azimuth of the optical axis of cervical tissue. These images highlighted both the inner structure of cervix and the arrangement of cervical collagen fibers confirmed by the second harmonic generation microscopy. The statistical analysis and two-Gaussians fit of the distributions of linear retardance and linear depolarization in the entire images of cervical tissue (without manual selection of the specific regions of interest) quantified the randomization of collagen fibers alignment with gestation time. At day 18 the remodeling of cervical extracellular matrix of collagen was measurable at the external cervical os that is available for the direct optical observations *in vivo*. It supports the assumption that imaging Mueller polarimetry holds promise for the fast and accurate collagen scoring in pregnancy and the assessment of the preterm birth risk.

## Introduction

Preterm birth (PTB) is a public health problem worldwide. PTB complications are the most important cause of death in neonatal infants, and many survivors will face long-term health challenges^1^. The accurate assessment of PTB risk is critical both to devise new treatment options as well as for the deployment of the available intervention focused on prolongating the pregnancy, such as cervical cerclage, pessaries, or special drug administration.

The two main functions of uterine cervix in pregnancy include: (1) maintain its load bearing capability and integrity in the first phase of pregnancy, thus, letting a fetus to develop properly until delivery time and (2) prepare to labor and delivery by cervical tissue softening through physical and chemical changes that are part of the cervix ripening process and will let the cervix dilate during delivery. The main constituent of the extracellular matrix (ECM) of cervical tissue is fibrillar collagen^2^. Several studies suggest that the evolution of mechanical properties of cervical tissue in pregnancy is related to cervix softening because of cervical ECM remodeling^3,4^.

All steps of the uterine cervix remodeling process are drastically accelerated in preterm labor^5,6^ leading to premature delivery. The early detection of the PTB risk may prevent this event and decrease both mortality and morbidity in infants as well as lowering health care system expenditures.

The ultrasonographic examination of cervix and the test of fetal fibronectin may help to avoid unnecessary treatment in case of negative results, but these techniques are not used for the PTB screening in pregnant women because of the low accuracy^7,8^. Despite the extensive preclinical studies^9–11^ there are no clinical tools available for the fast and accurate detection of a spontaneous PTB risk.

An extreme sensitivity of polarized light to the subtle alterations of the structural components of such complex object as biological tissue^12,13^ suggests exploring optical polarization for the assessment of the remodeling of cervical ECM in pregnancy. The development of polarization sensitive optical techniques for the accurate, fast, and non-contact diagnosis of the PTB risk in clinical settings represents the real challenge, as these modalities have potential to revolutionize the current medical practice of PTB risk diagnosis. The most promising approach includes using the complete Mueller polarimetry^14–16^ combined with the state-of-the-art Mueller matrix decompositions and data processing algorithms^17–20^, because (i) Mueller matrix images of a sample contain information on all polarimetric properties (diattenuation, retardance, depolarization)^14^ of a sample, contrary to the incomplete polarimetric techniques (e.g. Stokes polarimetry, orthogonal state contrast measurements, etc.), and (2) imaging Mueller polarimetry does not require sample scanning, all 16 Mueller matrix images of the entire cervix can be imaged in a few seconds an one wavelength^21,22^. Using Mueller polarimetric system in a visible wavelength range for the PTB risk assessment is a natural and safe choice, because visible light is harmless for the patients. Preliminary in vivo polarimetric studies of human uterine cervix in reflection configuration^22^ revealed linear birefringence of healthy cervical tissue; however, the detailed studies are needed to relate the observed optical anisotropy to the state of collagen ECM of uterine cervix during the pregnancy. Hence, we unavoidably face the fundamental questions:

- Is optical polarization sensitive to the modifications of collagen arrangement in the uterine cervix during pregnancy?
- Could the polarimetric signature of cervical collagen rearrangement be detected early enough to serve as a predictive marker of PTB risk despite shallow penetration depth of visible light in tissue because of strong scattering?

Our case study of mice model of pregnancy address these questions using the custom-built complete imaging Mueller polarimetric system^23^ operating in a visible wavelength range in transmission configuration.

## Results

Thin unstained sections of the upper and lower parts of the mouse uterine cervix from mice model of normal pregnancy at days 6 and 18 of a 19-days gestation period were imaged with the Mueller matrix polarimeter in transmission configuration. The experimentally recorded Mueller matrices were processed with the logarithmic Mueller matrix decomposition (LMMD) to obtain the images of polarization and depolarization parameters^14^. We focused our analysis on the images of the total linear retardance $R_L$, the linear depolarization $\alpha _{22}$ and the azimuth of the optical axis of linear uniaxial birefringent medium. There was no measurable diattenuation and circular retardance detected, the circular depolarization values were almost equal to the values of linear depolarization.

**Figure 1:**
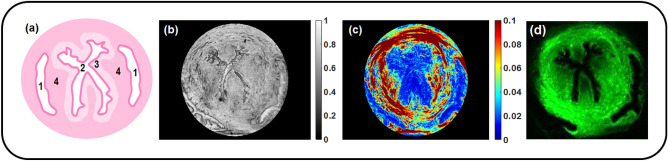
(**a**) The schematic of a transverse cross-section of the mouse uterine cervix: 1–vaginal fornix, 2–cervical canal, 3–subepithelial stroma, 4–midstroma. Unstained section of the upper part of a mouse cervix (6 days of gestation) measured with Mueller polarimeter: (**b**) total transmitted intensity (grey scale), (**c**) total linear retardance (rads), field of view (FoV) is 3 mm; (**d**) SHG microscopy image. The collagen fibers are shown in green, the brighter color corresponds to the higher concentration of the collagen fibers.

**Figure 2:**
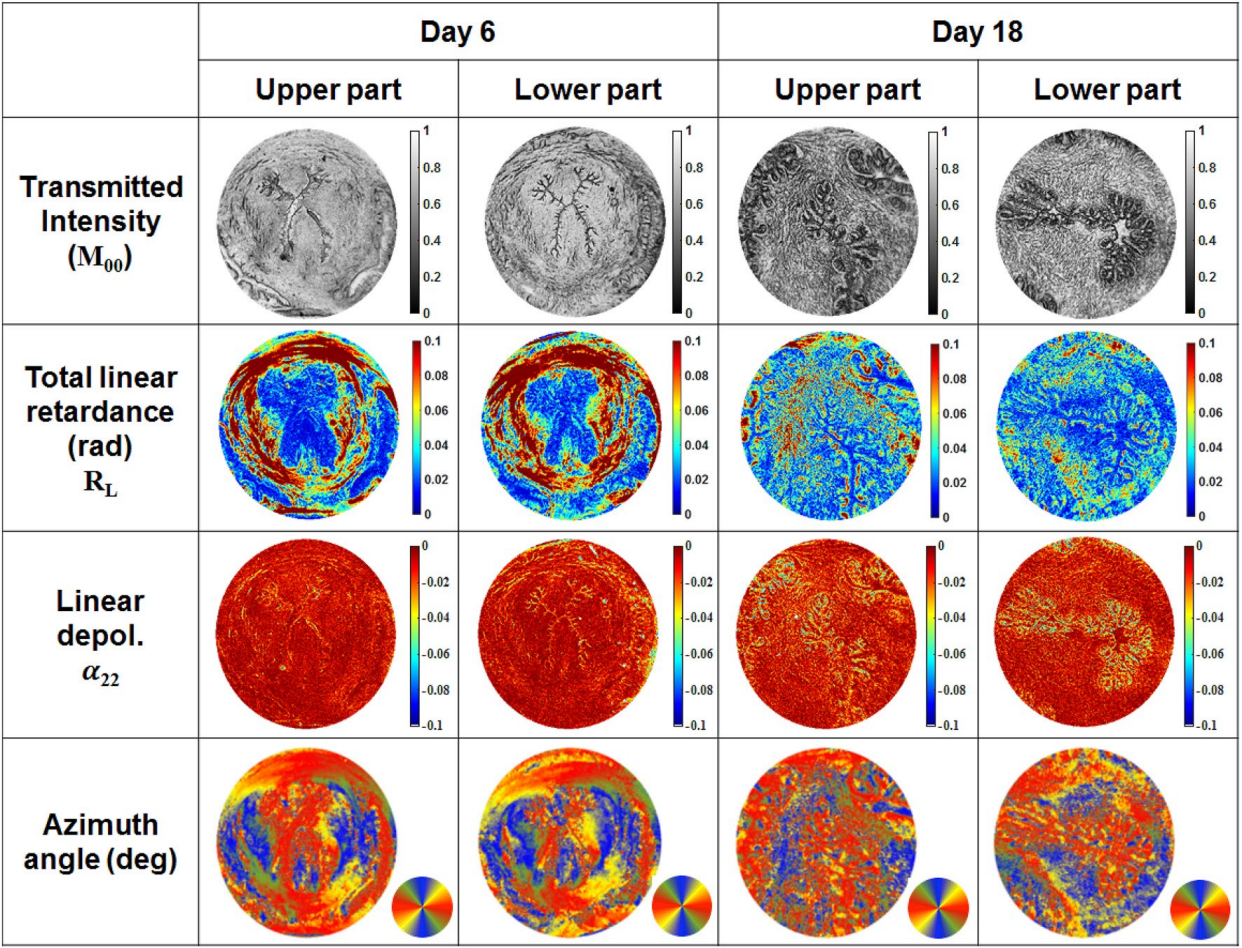
Images of the unstained tissue sections from both upper and lower parts of mice uterine cervices at 6 and 18 days of gestation; first row–total transmitted intensity (a.u.), second row–the total linear retardance $R_L$(rad), third row–the linear depolarization $\alpha _{22}$ (dimensionless), fourth row–the azimuth of the optical axis (degrees), circular color bar shows orientation in the imaging plane: red–vertical, blue-horizontal, green/yellow $\pm 45^\circ$, respectively.

The schematic of a transverse cross-section of the mouse uterine cervix is shown in Fig. 1a. The specific structural features include the X-shaped cervical canal, the zones of subepithelial stroma and midstroma as well as the vaginal fornix. We measured first the section of the upper part of the mouse cervix at day 6 of pregnancy with the imaging Mueller polarimeter (Fig. 1b,c). Then the same section of cervical tissue was measured with a non-linear second harmonic generation (SHG) microscopy (Fig. 1d). The grey scale total transmitted intensity image of this tissue section highlights some specific inner structures, like X-shaped cervical canal and vaginal fornix (Fig. 1b), but no contrast is observed between the subepithelial stroma and midstroma zones, whereas this contrast is clearly visible in the image of the total linear retardance. The high values of the total linear retardance (up to 0.1 radians) within the circumferential zone of midstroma (Fig. 1c) help to visualize a clear border of the low retardance subepithelial stroma around the X-shaped cervical canal. We attribute the high values of midstroma retardance to the strong anisotropy of refractive index of the densely packed collagen fibers (so called form birefringence). The circumferential arrangement of the collagen fibers is also confirmed with the SHG microscopy measurements^24^ (Fig. 1d) that is the gold standard technique for collagen visualization^25,26^. SHG microscopy has been used extensively to image the mouse cervix which is reach of collagen type 1. Several studies have demonstrated SHG capability to image collagen fibers showing increased collagen fibers diameters, less organization and more crimped fibers^9,27,28^. The set of the images of unstained sections of cervical tissue at day 6 of pregnancy from both upper and lower parts of the mouse uterine cervix is shown in the first two columns of Fig. 2.

The X-shaped cervical canal is clearly seen in the images of both total transmitted intensity and linear depolarization. The epithelium of cervical canal is more depolarizing compared to the subepithelial stroma and midstroma zones ($\alpha _{22} \le 0$, $\alpha _{22} = 0$ means no depolarization). This is most likely due to the composition of the cellular and ECM at this location with higher elastin content compared to collagen^18^.

**Figure 3:**
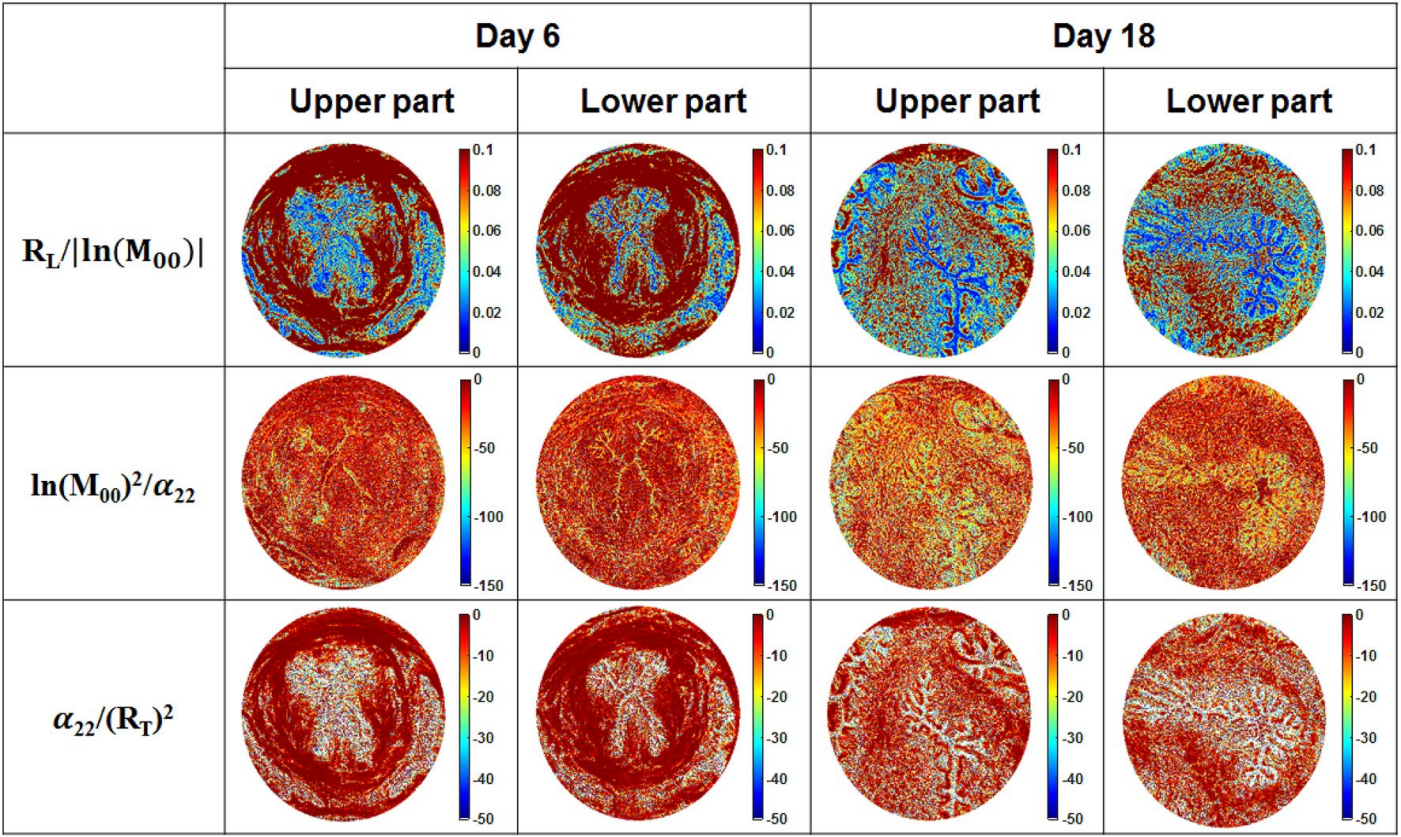
Normalized/fused images of the unstained tissue sections from the upper and lower parts of mice uterine cervices at 6 and 18 days of gestation. $M_{00}$ is the element of Mueller matrix of a sample representing the total transmitted intensity.

The total linear retardance images of the sections from upper and lower parts of the cervix (day 6) demonstrate circular arrangement of the collagen fibers in a midstroma around cervical canal (Fig. 2, second row). The zone of vaginal fornix has low values of linear retardance due to the absence of the aligned collagen fibers. However, the low values of total linear retardance within the subepithelial stroma as well as non-zero values of linear depolarization (Fig. 2, third row) indicate the different inner structure of this zone compared to the midstroma of cervical tissue. The collagen is still present in the subepithelial stroma, but the density and orientation of fibers are different from that of midstroma zone. The area of subepithelial zone is larger in the upper part of cervix compared to the lower part of cervix at day 6 of pregnancy. It confirms that the cervix ripening process starts from the internal os towards the external os^29–31^. The capability of Mueller polarimetry to visualize collagen at a micrometric scale was already shown in the images of thin sections of human cervical and vaginal tissue^32,33^, whereas our experiments demonstrate that imaging Mueller polarimetry provides a polarimetric signature of collagen at a mesoscopic scale of a few mm without doing sample scanning. The normalized/fused images of cervical tissue sections from the upper and lower parts of cervix (day 6 of pregnancy) are shown in Fig. 3 (first and second columns). We applied the procedure of normalization/fusion pixelwise to exclude the impact of tissue thickness fluctuations. The images of normalized linear retardance at day 6 of pregnancy (Fig. 3, first row) confirm the circumferential arrangement of midstroma collagen around the cervical canal. The normalized linear retardance image of the section of lower part of cervix demonstrates additional contrast between the subepithelial stroma and epithelium of cervical canal that was not seen in the corresponding nonnormalized image of the total linear retardance (Fig. 2, second row). The fine contours of the cervical canal are contrasted in the images of the reciprocal normalized linear depolarization (Fig. 3, second row). The fused images of the total linear retardance and the linear depolarization (Fig. 3, third row) highlight the anatomical structure of mouse cervical tissue by increasing the contrast between the midstroma, the subepithelial stroma and the epithelium of cervical canal for both upper and lower sections of cervix at day 6 of pregnancy. We have shown here that imaging Mueller polarimetry is sensitive to the arrangement of cervical collagen and the polarimetric images demonstrate increased contrast between different structural zones of cervical tissue.

## Discussion

Now we address the problem of assessing the capability of the imaging Mueller polarimetry to become a clinical tool for in vivo detection of the PTB risk. Visible light does not penetrate deep in strongly scattering biological tissues (like uterine cervix) compared to the signals of ultrasound and MRI modalities^11,34^. The optical clearing approach helps to increase the penetration depth of light considerably by dumping tissue scattering^35^. However, the development of a non-contact imaging modality that does not require an application of any chemical agent for the image contrast increase for the PTB risk assessment in humans will be a significant breakthrough.

**Figure 4:**
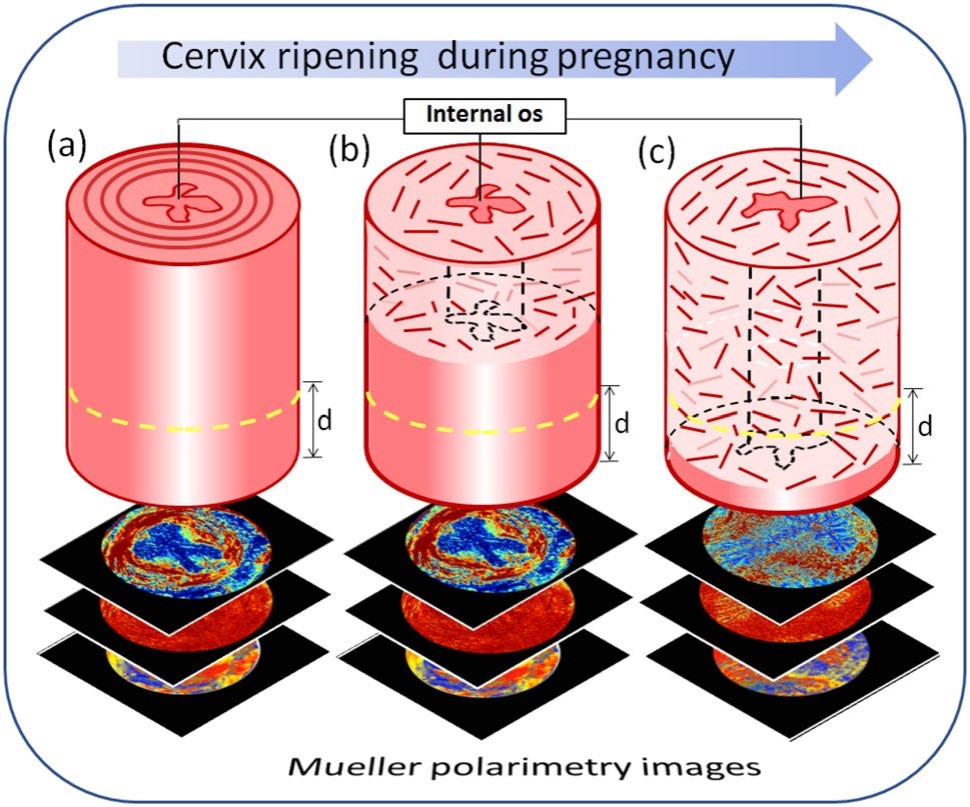
Schematic of the evolution of the cervical ECM in pregnancy that starts from the internal os region and progresses towards an external cervical os: (**a**)–the first day of pregnancy; (**b**) mid-term; (**c**) the day of pregnancy when the remodeling of cervical ECM collagen can be detected with a noncontact imaging Mueller polarimeter in reflection configuration. Visible light impinges on the lower part of cervix and d denotes its penetration depth (yellow dashed line).

It was shown that the wide-field imaging Mueller polarimetry in backscattering configuration provides high contrast images and highlights tissue microarchitecture^22,36^ or pathological zones^37–40^ that are not visible in the unpolarized intensity images. An imaging Mueller polarimeter integrates the signal over the volume of the probed tissue. This volume is defined by the thickness of tissue slab when measurements are performed in transmission configuration and by light signal penetration depth for the measurements in backscattering configuration. The latter is the most relevant optical measurement geometry for *in vivo* medical applications. Cervical ECM remodeling starts from the internal os towards the external os, the latter can be examined by a medical doctor during the colposcopy test^41^. The question arises – at which moment of pregnancy the ECM remodeling and consequent softening of cervix can be detected by an optical technique operating within the visible wave band (harmless for a patient) and having shallow penetration depth? Will the risk of PTB be detected early enough to allow for the deployment of necessary treatment to prevent PTB? The illustration of this problem is shown in Fig. 4.

**Figure 5:**
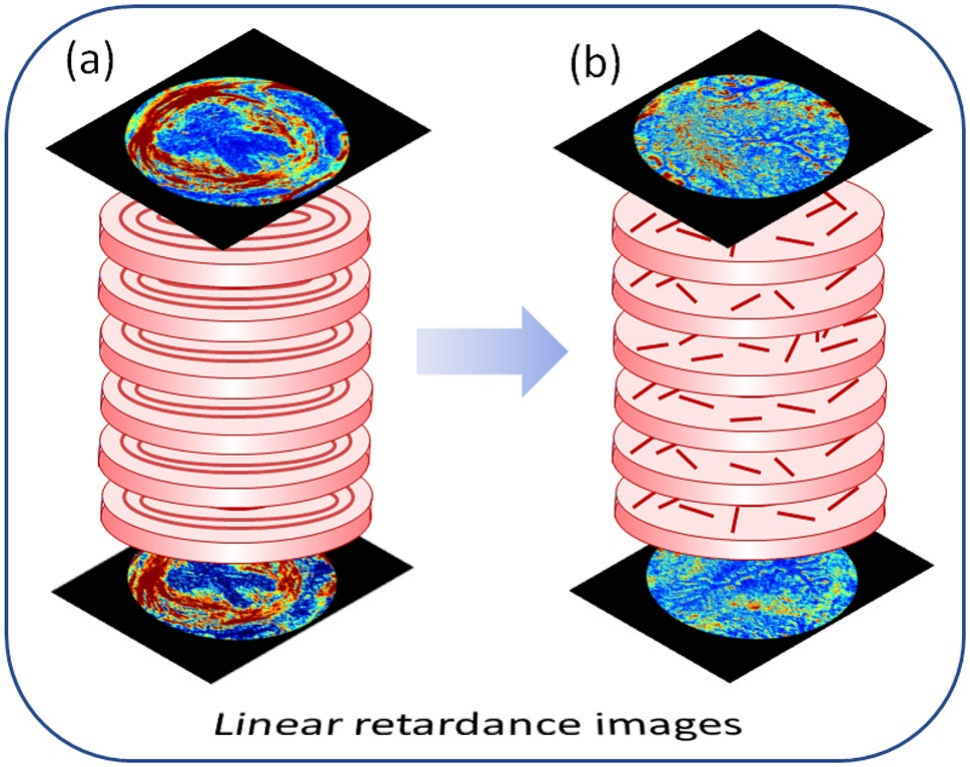
Schematics of the collagen ECM remodeling through the mouse cervix from (**a**) day 6 to (**b**) day 18 of pregnancy as seen in the images of total linear retardance of the upper and lower sections of the cervices. One day before delivery the ECM of collagen of entire cervix is already remodeled in mice model of pregnancy.

The images of tissue sections from both upper and lower parts of the mouse uterine cervix at day 18 of pregnancy are shown in the third and fourth columns of Figs. 2 and 3. The epithelial layer of cervical canal becomes thicker and more depolarizing as seen in the images of linear depolarization (Fig. 2, third row) and reciprocal of the normalized linear depolarization (Fig. 3, second row). A complete loss of circular arrangement of the collagen fibers around the cervical canal in midstroma zone is observed for both upper and lower sections of cervix one day before the delivery in the images of the non-normalized and the normalized total linear retardance. The area of subepithelial stroma increased in the latter images compared to the corresponding images at day 6 for both upper and lower cervical sections. The maps of the azimuth of the optical axis demonstrate a completely random orientation of collagen fibers in the images of both upper and lower cervical sections at day 18 of the pregnancy. This means that the remodeling process of the cervical ECM has affected the entire cervix (Fig. 5).

It confirms that the remodeling of ECM of collagen induced by cervix ripening can be detected with imaging Mueller polarimetry at least one day prior to delivery at day 19 in mice model of normal pregnancy.

**Figure 6:**
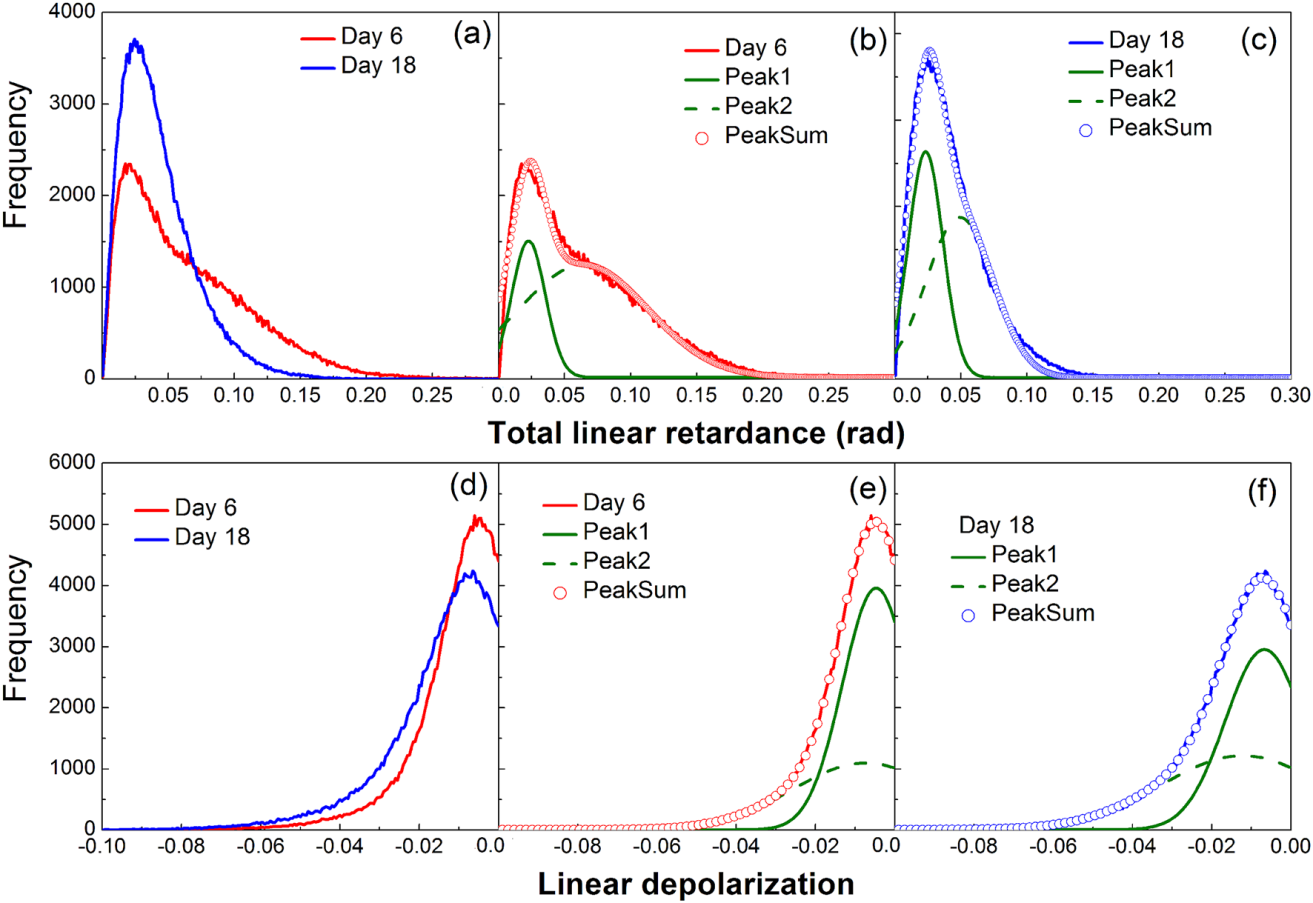
Histograms for the polarimetric images of the lower sections of mice cervices (**a**) total linear retardance at day 6 and day 18; (**b**) two-Gaussians fit of the histogram of the total linear retardance at day 6; (**c**) two-Gaussians fit of the histogram of the total linear retardance at day 18; (**d**) linear depolarization at day 6 and day 18 of pregnancy; (**e**) two-Gaussians fit of the histogram of the linear depolarization at day 6; (**c**) two-Gaussians fit of the histogram of the linear depolarization at day 18.

For the quantitative cervical collagen scoring during the course of pregnancy we conducted a statistical analysis of the distributions of the polarimetric parameters. The histograms of total linear retardance and linear depolarization in the images of the sections of lower part of mice cervices at days 6 and 18 of pregnancy are shown in Fig. 6.

The mean values of the total linear retardance are relatively small (see Table 1), because we did not perform any manual selection of the regions of interest rather averaging was performed over the entire image of tissue sections, including the zones of cervical os, subepithelial stroma and vaginal fornix with the vanishing values of retardance.

**Table 1:** Statistical moments of the distributions of total linear retardance and linear depolarization in the images of sections of lower part of uterine cervix at different gestation time points.

Parameter	Gestation day	Mean value (rad)	Standard deviation	Skewness	Kurtosis
Linear retardance	D6	0.067	0.041	1.35	0.66
	D18	0.042	0.021	2.04	2.83
Linear depolarization	D6	− 0.013	0.008	2.84	6.85
	D18	− 0.017	0.010	2.33	4.07

However, the mean value of retardance is higher at day 6 compared to the corresponding value at day 18. The value of standard deviation for $R_L$ at day 6 is twice of that at day 18. The pronounced difference between the distributions of the total linear retardance values at day 6 and day 18 is clearly seen in the values of skewness and kurtosis.

The fit of the histograms of retardance at day 6 and day 18 with two Gaussian distributions (Fig. 6b,c) confirms the presence of two different sets of pixels. The circumferential zone of arranged collagen fibers with high values of retardance (day 6) is not present in the image of cervical tissue section at day 18. With cervix ripening the optically anisotropic zones of densely packed and aligned collagen fibers are gradually transformed into the optically isotropic ones because of cervical ECM remodeling. Thus, the position of the peak and the width of the second Gaussian distribution for the histogram of retardance hold promise for the accurate collagen scoring during pregnancy.

The mean values of linear depolarization are close to zero ($\alpha _{22}=0$ for non-depolarizing sample) for both cervical sections at day 6 and 18 (Fig. 6d), whereas the latter is slightly more depolarizing (see Table 1). The scattering of light is not strong in thin tissue sections, so the depolarization of incident polarized light is low. The values of standard deviation for the linear depolarization at day 6 and 18 are also close. The difference between the distributions of the linear depolarization values at day 6 and 18 is more pronounced in the corresponding values of skewness and kurtosis. As in the case of total linear retardance the sum of two contributions is confirmed for the linear depolarization values by fit with two Gaussian distributions of the corresponding histograms at day 6 and 18 (Fig. 6e,f).

**Figure 7:**
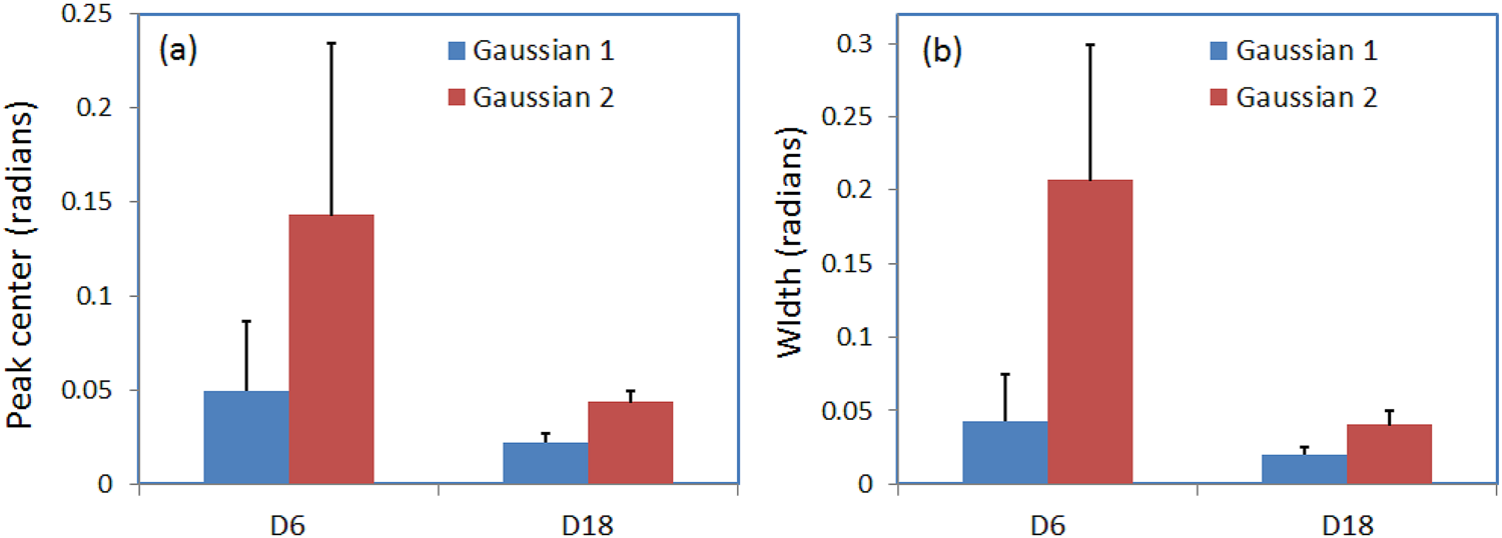
Statistical analysis with two-Gaussian fit of retardance distributions in the images of lower uterine sections (five mice at day 6 and three mice at day 18); (**a**) mean value and standard deviation of (**a**) center peak and (**b**) the width of both Gaussian distributions.

The statistical analysis of the scalar retardance and the depolarization images of lower sections of uterine cervix taken from five mice at day 6 and three mice at day 18 of pregnancy was performed using two-Gaussian fit. The position and width of both first and second peaks for both retardance and depolarization distributions were calculated and averaged over each pregnancy time group. The statistical analysis of depolarization did not reveal the correlation with pregnancy time. The mean value and standard deviation of the center and width of the first and second peaks for retardance distributions are shown in Fig. 7. The average values of center of both first and second peaks decrease with time of pregnancy, however, the difference is more pronounced for the center of the second peak. Quite large values of standard deviation for the center of both peaks at day 6 reduce significantly at day 18 (Fig. 7a). It reflects the loss of optical anisotropy of cervical tissue during cervix remodeling before the delivery. The same trends with time of pregnancy are observed for the averaged value and standard deviation of the width of both Gaussian distributions.

It is worth mentioning that the path length of the detected light may become larger for the measurements of thick tissue in reflection geometry, especially with a proper choice of the wavelength of incident light. It is known that red light penetrates deeper in live tissue compared to the shorter wavelengths in the visible wave band because of the maximum of hemoglobin absorption in a green part of spectrum. Using longer wavelength will change the depolarization values and the associated statistical parameters that can be used together with the corresponding values of linear retardance for the quantitative assessment of the softening of cervical ECM matrix and cervical collagen scoring.

The kurtosis of distribution of the azimuth of the optical axis was shown to be a promising metrics for the assessment of collagen arrangement in different zones of the cervix at different gestation time^22^. In this study we intentionally avoid any manual selection of the regions of interest (ROI) and analyzed the distributions of all polarimetric parameters over the whole image, because such approach removes any operator-dependent bias in selecting the ROIs. In such case the parameters of circular statistics of the azimuth distribution show no significant difference between day 6 and day 18 of pregnancy, because the circularly arranged collagen fibers of midstroma at day 6 are covering the range of azimuth angles from 0 degrees to 360 degrees as well as the randomly distributed collagen fibers at day 18.

The polarimetric measurements of tissue in backscattering geometry will inevitably deal with the specular reflection of incident light on the uneven surface of tissue that will affect the backscattered signal. Such image pixels, however, can easily be detected and omitted from the analysis, because of a low depolarization of light specularly reflected by a surface^42^.

The suggested metrics for collagen scoring by the analysis of mean value, standard deviation, skewness and kurtosis of the distributions of retardance and depolarization in the images of cervical tissue as well as the parameters of fit with two Gaussian distributions do not require any image segmentation and selection of the special regions of interest for data analysis, and contain the valuable information on remodeling of the cervical ECM of collagen that may serve for the prediction of PTB risk.

We have demonstrated the capability of a new promising polarimetric imaging technique, namely, imaging Mueller polarimetry operating in a visible wavelength range to detect the cervical ripening in mice model of normal pregnancy. We showed that cervical ripening starts from the internal os and progresses towards the external os of cervix. The state of cervical ECM remodeling is not detectable in the standard intensity images but is highly contrasted in the images of the total linear retardance and the azimuth of the optical axis. The circumferential arrangement of midstroma collagen around the cervical canal and subepithelial stroma zone was seen in the above mentioned images and confirmed by the SHG microscopy measurements.

The polarimetric images of cervical tissue sections at day 18 confirmed the remodeling of cervical ECM throughout the entire cervix and, thus, complete ripening of mouse uterine cervix one day before delivery at day 19. As it was mentioned the PTB is characterized by the drastic acceleration of the normal process of cervix ripening. We demonstrated that the remodeling in cervical collagen affected the entire cervix at least one day before delivery in mice model of pregnancy. Hence, this remodeling can be detected with the imaging Mueller polarimetry in reflection geometry (that is relevant for clinical applications) as well, despite the fact that the penetration depth of Mueller polarimetry does not exceed couple of millimeters in cervical tissue in a visible wavelength range.

The most informative collagen scoring metrics include the statistics on the total linear retardance and the linear depolarization images of cervical tissue that were calculated with the logarithmic decomposition of the recorded Mueller matrices. Various Mueller matrix decompositions are available for the matrices recorded in backscattering configuration^17^, and suggested collagen scoring metrics can be applied for the distributions of the retardance and depolarization values in the corresponding images of uterine cervix recorded *in vivo*.

Whereas the mouse reproductive system is different from that of human, the evolution of ECM of cervix during pregnancy in mice is representative of the same process in humans, but with a shorter time scale. It supports our assumption that Mueller polarimetry has potential to become a technique of choice for the fast, accurate and non-contact cervical collagen scoring and quantitative *in vivo* assessment of the PTB risk in humans during the colposcopy test^22^.

## Methods

### Preparation of the sections of mouse uterine cervix

The mouse strain C57BL6/129sv mice were used and pregnant females in this study were between 3-6 months of age. For timed pregnancies, breeding pairs were set up in the morning for 6 hours. The presence of a vaginal plug at the end of the 6 hours period was considered day 0 of pregnancy. The birth of pups generally occurred in the early morning on day 19.

Mice were housed in an approved animal resource facility. All animal procedures were performed in accordance with the standards of humane animal care following the NIH Guide for the Care and Use of Laboratory Animals. The research protocols were reviewed and approved by the Institutional Animal Care and Use Committee at the University of Texas Southwestern Medical Center (registration number: IACUC 2016-101519) and at Florida International University (registration number: IACUC-20-014). All animals were maintained and used in accordance with the ARRIVE guidelines.

**Figure 8:**
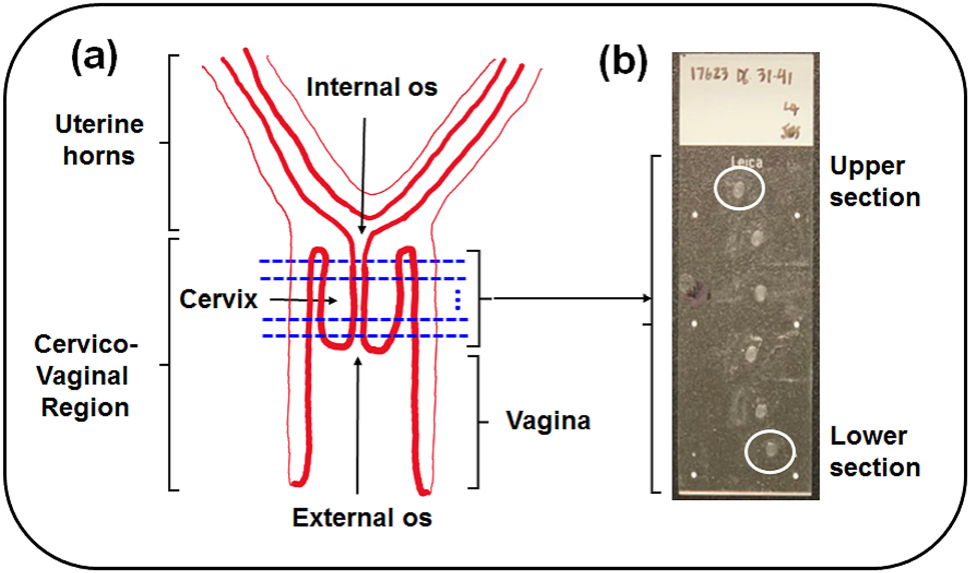
Illustration of the cervical tissue sectioning procedure: (**a**) schematic longitudinal cross-section of a lower part of mouse reproductive tract. The location of transverse planes of the cervix cuts is shown by the blues dashed lines; (**b**) photo of the microscope glass slide with the cervical tissue sections (day 6 of pregnancy). The imaged upper (internal os) and lower (external os) sections of uterine cervix are highlighted by the white circles.

In normal mouse pregnancy the remodeling of cervical ECM starts between day 6 and day 12 of a 19-days gestation period^43–45^. The mouse uterine cervix consists of the connective tissue mainly. It has a cylindrical shape, and there is an inner cervical canal that connects both the uterus and vagina (Fig. 8a). The length of cervix is about 2.8 mm in non-pregnant mice, and it increases up to 4.4 mm at the end of 19-days gestation period. The uterine cervices used in the polarimetric studies were obtained from eight pregnant mice, including five at gestation day 6 and three at gestation day 18.

First the mice cervices were snapped frozen at − 80° C in optimal cutting temperature compound (Tissue Tek, Elkhart, Indiana). A cryostat (Leica CM3050) was used for the transverse cryosectioning of the whole length of cervix (Fig. 8a) at − 20 °C. The sections of cervical tissue with a nominal thickness of 50 μm were mounted on the glass slides and kept 1 hour at room temperature for drying (Fig. 8b). The slides without a cover slip were used.

### Imaging Mueller matrix polarimeter in transmission configuration

**Figure 9:**
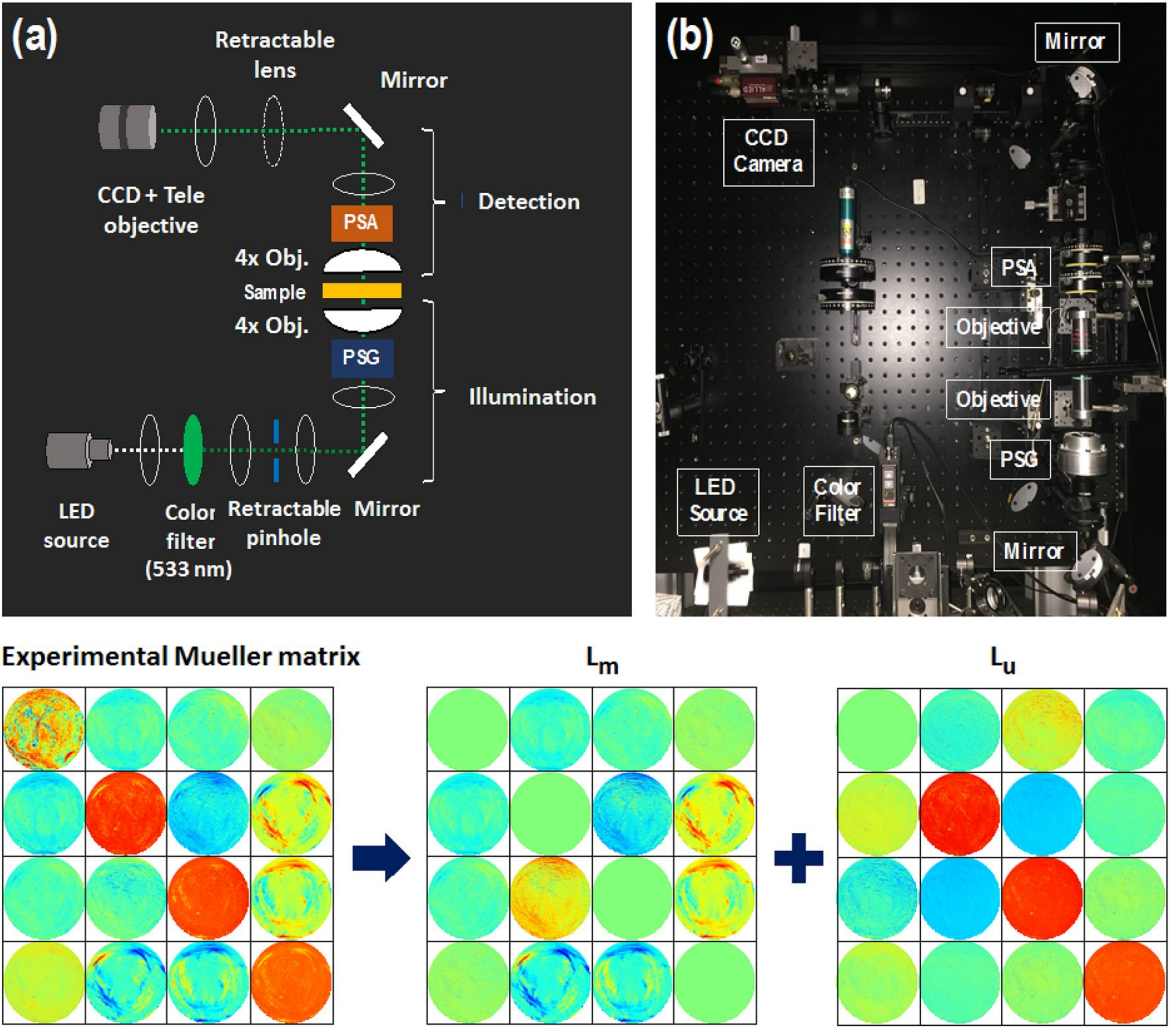
Imaging Mueller polarimeter: (**a**) schematic layout, (**b**) photo of the instrument. Bottom row, left to right: measured Mueller matrix images (FoV 3 mm) of mouse cervical tissue section, the matrices $\mathbf {L_m}$ and $\mathbf {L_u}$ are obtained with the logarithmic decomposition of experimental Mueller matrix. The elements of the matrices $\mathbf {L_m}$ and $\mathbf {L_u}$ have a straightforward physical interpretation in terms of the polarimetric properties of a sample. The values of all matrix coefficients vary between -0.2 (blue) to 0.2 (red) except the values of $M_{00}$ coefficient that vary from 0 (blue) to 1 (red), and the values of diagonal elements of $\mathbf {L_u}$ that vary from − 0.1 (blue) to 0 (red).

The custom-built liquid crystal-based Mueller polarimetric imaging system^23^ operating in a visible wavelength range was used to measure the Mueller matrices of the sections of mouse uterine cervices in transmission configuration (Figs. 9a and 9b). The wavelength of 533 nm was selected for our measurements by placing an interferential filter (20 nm spectral bandwidth) after the white light LED source (Stemmer Imaging, Germany). Four different polarization states of the illumination light beam were generated by the polarization state generator (PSG). The PSG consists of a linear polarizer, two ferroelectric liquid crystal retarders (Meadowlark FPR-200-1550, USA), and a quarter-wave retarder. Two objective lenses (Nikon CFI LU Plan Fluor, 4X, Japan) were placed above and below the imaging plane with a field of view (FoV) about 3 mm. The light beam emerging from a sample passed through the polarization state analyzer (PSA) that comprises the same components as those of the PSG with a reverse order of arrangement. Finally, the light beam reaches the CCD camera (AV Stingray F-080B, Allied Vision, Germany, image resolution 600 × 800 pixels) that is coupled to a telephoto lens with a focus to infinity. The calibration of the instrument was performed with the eigenvalue calibration method described elsewhere^46^. Sixteen measurements (four different polarization states from the PSG are sequentially projected on the same four polarization states from the PSA) were performed to calculate sixteen coefficients of a sample Mueller matrix for each image pixel. Non-linear SHG microscopy images were obtained with the SAMMM system^24^.

### Logarithmic decomposition of Mueller matrix

Several algorithms of nonlinear data compression were proposed for the physical interpretation of Mueller matrix data^17,47^. Among all algorithms a logarithmic decomposition LMMD developed for the transmission measurements considers all optical properties as continuously distributed through the volume of a medium along the path length of probing beam. It makes LMMD particularly suitable for the studies of biological tissues. The key steps of LMMD are briefly recalled below. Within the framework of differential matrix formalism of a fluctuating anisotropic medium^48–51^ the transmission Mueller matrix is described by the following equation:

1 $$\begin{aligned} \frac{d{\mathbf {M}}(z)}{dz} ={\mathbf {m}}{\mathbf {M}}(z) \end{aligned}$$

Mueller matrix ${\mathbf {M}}(z)$ that depends on the optical path length *z*, is associated with a unique differential matrix ${\mathbf {m}}$. This differential matrix is constant for both non-depolarizing and depolarizing media that are homogeneous along the light beam direction. For a depolarizing medium, the differential matrix **m** can be decomposed into a sum of matrices $\mathbf {L_m}$ and $\mathbf {L_u}$:

2 $$\begin{aligned}&{\mathbf {m}}=\mathbf {m_m}+\mathbf {m_u},\quad \quad \mathbf {m_m}=\frac{1}{2}({\mathbf {m}}-{\mathbf {G}}\mathbf {m^T}{\mathbf {G}}),\quad \quad \mathbf {m_u}=\frac{1}{2}(\mathbf{m }+{\mathbf {G}}\mathbf {m^T}{\mathbf {G}}) \end{aligned}$$

3 $$  {\mathbf{m}}_{{\mathbf{m}}}  = \left[ {\begin{array}{*{20}l}    0 \hfill & {p_{1} } \hfill & {p_{2} } \hfill & {p_{3} } \hfill  \\    {p_{1} } \hfill & 0 \hfill & {p_{6} } \hfill & { - p_{5} } \hfill  \\    {p_{2} } \hfill & { - p_{6} } \hfill & 0 \hfill & {p_{4} } \hfill  \\    {p_{3} } \hfill & {p_{5} } \hfill & { - p_{4} } \hfill & 0 \hfill  \\   \end{array} } \right],\;{\mathbf{m}}_{{\mathbf{u}}}  = \left[ {\begin{array}{*{20}l}    {d_{0} } \hfill & {d_{1} } \hfill & {d_{2} } \hfill & {d_{3} } \hfill  \\    { - d_{1} } \hfill & {d_{0}  - d_{7} } \hfill & {d_{6} } \hfill & {d_{5} } \hfill  \\    { - d_{2} } \hfill & {d_{6} } \hfill & {d_{0}  - d_{8} } \hfill & {d_{4} } \hfill  \\    { - d_{3} } \hfill & {d_{5} } \hfill & {d_{4} } \hfill & {d_{0}  - d_{9} } \hfill  \\   \end{array} } \right]  $$

where matrix ${\mathbf {G}}=diag(1,-1,-1,-1)$ is the Minkowski metric and T denotes matrix transposition. The elements of matrix $\mathbf {m_m}$ represent the linear $(x-y)$ dichroism $p_1$, linear $(\pm 45^{\circ })$ dichroism $p_2$, and circular dichroism $p_3$, linear $(x-y)$ retardance $p_4$, linear $(\pm 45^{\circ })$ retardance $p_5$, and circular retardance $p_6$. The elements of $\mathbf {m_u}$ describe the depolarization properties of the medium. Diagonal terms $d_7$, $d_8$ and $d_9$ represent the anisotropic depolarization coefficients, the off-diagonal elements show the uncertainties of the corresponding polarization properties. For a continuous depolarizing medium that is homogeneous in the direction of light propagation the following relation holds^52^

4 $$\begin{aligned} {\mathbf {M}}(z)=\exp {[\langle {\mathbf {m}}\rangle z+ \frac{1}{2}\langle \Delta {\mathbf {m}}^2\rangle z^2]} \end{aligned}$$

where the matrix $\mathbf {m_m}=\langle {\mathbf {m}} \rangle$ presents the mean values of the polarization properties, whereas the matrix $\mathbf {m_u}=\langle \Delta {\mathbf {m}}^2 \rangle z$ contains their uncertainties and linearly depends on the slab’s thickness *z*. Spatial averaging is performed in the transverse plane to the direction of light propagation. It follows from Eq. (4) that the mean values of the polarimetric properties scale up linearly with the slab thickness while the depolarization properties evolve quadratically with it. We will explore this dependence for the increase contrast in polarimetric images.

To obtain the differential matrix ${\mathbf {m}}$ of a homogeneous medium from the experimental Mueller matrix M of a sample one needs to compute the matrix logarithm, which can be represented as a sum of two matrices $\mathbf {L_m}$ and $\mathbf {L_u}$ of opposite ${\mathbf {G}}$-symmetry (Fig. 9, bottom panel).

5 $$  {\mathbf{L}} = \ln {\mathbf{M}},\;{\mathbf{L}} = {\mathbf{L}}_{{\mathbf{u}}}  + {\mathbf{L}}_{{\mathbf{m}}}   $$

6 $$\begin{aligned}&\mathbf {L_m}=\frac{1}{2}({\mathbf {L}}-{\mathbf {G}}\mathbf {L^T}{\mathbf {G}}),\quad \quad \mathbf {L_u}=\frac{1}{2}({\mathbf {L}}+{\mathbf {G}}\mathbf {m^T}{\mathbf {G}}) \end{aligned}$$

Calculating the logarithm of Eq. (4) at $z = 1$ we observe that the matrices $\mathbf {L_m}$ and $\mathbf {L_u}$ equal the mean values and (half) the variances of the polarization properties, respectively, that accumulated over the slab thickness:

7 $$\begin{aligned} \mathbf {L_m}=\mathbf {m_m} =\langle {\mathbf {m}} \rangle , \quad \mathbf {L_u} = \frac{1}{2}\mathbf {m_u}= \frac{1}{2}\langle \Delta \mathbf {m^2} \rangle \end{aligned}$$

### Normalization and fusion of polarimetric data

The optical anisotropy, scattering and absorption properties of tissue as well as the path length of the detected light beam that travelled through tissue will all affect the measured values of tissue polarization and depolarization parameters. When a thin tissue section is measured in transmission the path length of the detected light beam is equivalent to sample thickness. Thus, the thickness of tissue section will also impact the polarization and depolarization parameters calculated with LMMD from the experimental Mueller matrices. We suggest mitigating the spatial fluctuations of thickness of tissue section in a transverse plane by exploring Eq. 4. We denote by $R_L=\sqrt{p_4^2+p_5^2 }$ the total linear retardance that is invariant under the rotation of a sample within the imaging plane, and by $\alpha _{22}$ - the linear (x-y) depolarization parameter (i. e. the diagonal element $d_0-d_7$ of the matrix $\mathbf {L_u}$. Due to the linear dependence of $R_L$ values and the quadratic dependence of $\alpha _{22}$ values on sample thickness *z*, the following relations hold^23^:

8 $$\begin{aligned} R_L=A\cdot z, \quad \alpha _{22}=B \cdot z^2 \end{aligned}$$

where *A* and *B* are the coefficients that do not depend directly on a local thickness of sample. It follows from the Beer-Lambert law that $\ln {(I/i_0)}=-\mu _T \cdot z=\ln {M_{00}}$, where $I_0$ is an intensity of incident light, *I* - an intensity of transmitted light, $\mu _T=\mu _s+\mu _a$ is a sum of absorption coefficient $\mu _a$ and scattering coefficient $\mu _s$ of a medium, *z* is physical thickness of a sample. Consequently, the ratio of the parameters described below should not depend directly on a local thickness z:

9 $$\begin{aligned} \frac{R_L}{|\ln {(M_{00})}|}=\frac{A}{\mu _T},\quad \frac{\ln ^2{(M_{00})}}{\alpha _{22}}=\frac{\mu _T^2}{B},\quad \frac{\alpha _{22}}{R_L^2}=\frac{B}{A^2} \end{aligned}$$

Applying Eq. (9) pixel-wise we obtain a set of normalized/fused images of a sample. As the values of $\mu _T$, *A*, and *B* parameters may vary within the $(x-y)$ imaging plane, these images are not necessarily uniform. However, these spatial variations are related to the changes in tissue optical properties, and not to the changes in tissue thickness. First we applied the LMMD of experimental matrices and then data normalization/fusion algorithms for the increase of contrast in polarimetric images of cervical tissue sections. It is worth noting that the images obtained from Eq. (9) can be interpreted in terms of the normalized linear retardance, reciprocal to the normalized linear depolarization, and fused polarization-depolarization properties of a sample.
